# Comparative evaluation of the extracellular production of a polyethylene terephthalate degrading cutinase by *Corynebacterium glutamicum* and leaky *Escherichia coli* in batch and fed-batch processes

**DOI:** 10.1186/s12934-024-02547-2

**Published:** 2024-10-10

**Authors:** Stefanie Fritzsche, Holger Hübner, Marco Oldiges, Kathrin Castiglione

**Affiliations:** 1 Institute of Bioprocess Engineering, Department of Chemical and Biological Engineering, Friedrich-Alexander-Universität Erlangen-Nürnberg, Paul-Gordan-Straße 3, 91052 Erlangen, Germany; 2 Institute of Bio- and Geosciences, IBG-1: Biotechnology, Forschungszentrum Jülich GmbH, Wilhelm-Johnen-Straße, 52428 Jülich, Germany; 3 Institute of Biotechnology, RWTH Aachen University, Worringerweg 3, 52074 Aachen, Germany

**Keywords:** Enzymatic depolymerization, Polyethylene terephthalate, Recombinant protein expression, Extracellular, Secretion, Cutinase, Autoinduction, Bioreactor, Lactose feed

## Abstract

**Background:**

With a growing global population, the generation of plastic waste and the depletion of fossil resources are major concerns that need to be addressed by developing sustainable and efficient plastic recycling methods. Biocatalytic recycling is emerging as a promising ecological alternative to conventional processes, particularly in the recycling of polyethylene terephthalate (PET). However, cost-effective production of the involved biocatalyst is essential for the transition of enzymatic PET recycling to a widely used industrial technology. Extracellular enzyme production using established organisms such as *Escherichia coli* or *Corynebacterium glutamicum* offers a promising way to reduce downstream processing costs.

**Results:**

In this study, we compared extracellular recombinant protein production by classical secretion in *C. glutamicum* and by membrane leakage in *E. coli*. A superior extracellular release of the cutinase ICCG_DAQI_ was observed with *E. coli* in batch and fed-batch processes on a litre-scale. This phenomenon in *E. coli*, in the absence of a signal peptide, might be associated with membrane-destabilizing catalytic properties of the expressed cutinase. Optimisations regarding induction, expression temperature and duration as well as carbon source significantly enhanced extracellular cutinase activity. In particular, in fed-batch cultivation of *E. coli* at 30 °C with lactose as carbon source and inducer, a remarkable extracellular activity (137 U mL^−1^) and cutinase titre (660 mg L^−1^) were achieved after 48 h. Literature values obtained with other secretory organisms, such as *Bacillus subtilis* or *Komagataella phaffii* were clearly outperformed. The extracellular ICCG_DAQI_ produced showed high efficacy in the hydrolysis of PET textile fibres, either chromatographically purified or unpurified as culture supernatant. In less than 18 h, 10 g L^−1^ substrate was hydrolysed using supernatant containing 3 mg cutinase ICCG_DAQI_ at 70 °C, pH 9 with terephthalic acid yields of up to 97.8%.

**Conclusion:**

Extracellular production can reduce the cost of recombinant proteins by simplifying downstream processing. In the case of the PET-hydrolysing cutinase ICCG_DAQI_, it was even possible to avoid chromatographic purification and still achieve efficient PET hydrolysis. With such production approaches and their further optimisation, enzymatic recycling of PET can contribute to a more efficient and environmentally friendly solution to the industrial recycling of plastics in the future.

**Supplementary Information:**

The online version contains supplementary material available at 10.1186/s12934-024-02547-2.

## Background

In today’s world, the widespread use of plastics such as polyethylene terephthalate (PET) is a symbol of both human innovation and convenience. Unfortunately, this convenience comes at a substantial environmental cost, as PET’s exceptional durability leads to persistent accumulation in the environment, threatening global ecosystems [1, 2]. However, it is precisely this high chemical, physical and biological resistance, along with properties such as excellent strength-to-weight ratio, transparency and low production costs, that has led to the widespread use of PET, including disposable packaging and durable textile materials [3, 4]. Fortunately, enzymatic PET recycling emerged as a biotechnological answer to the search for a degradation process from PET to the recyclable monomers—terephthalic acid and ethylene glycol. Nowadays, a variety of PET-hydrolysing enzymes are known from a large number of organisms that already have naturally good or engineered properties in terms of catalytic activity and stability [5–9]. This, together with the optimisation of reaction conditions, now enables complete hydrolysis of post-industrial or post-consumer PET materials in less than 24 h, and efforts are underway for large-scale industrial applications [9, 10]. However, challenges remain in the efficient production of the involved biocatalysts, with downstream processing being a significant limitation, accounting for 50–70% of the total costs of most large-scale bioprocesses [11–13]. Therefore, the development of cost-effective expression systems is essential to meet the growing demand for PET degrading enzymes and to enable competitiveness with other plastics recycling methods. For instance, the use of protein secreting organisms can be a promising approach to minimise the costs and losses associated with cell disruption and subsequent processing. Certain organisms, such as *Ideonella sakaiensis* producing PETase and MHETase, naturally secrete PET degrading enzymes, though at low levels [14, 15]. So far, extracellular recombinant expression of cutinases has been successfully demonstrated in various expression systems. In *Bacillus subtilis* and *Komagataella phaffii*, secretion systems using different signal peptides have been effectively used to secrete cutinases to the extracellular environment [16, 17]. Also *C. glutamicum* is commonly used in combination with a variety of possible signal peptides to secrete recombinant cutinases via the Sec or Tat secretion pathway [18–20]. In *Escherichia coli*, extracellular release of cutinases has been observed both with and without the use of signal peptides without a targeted secretion pathway, presumably caused by a destabilization of membrane integrity [21–23]. In addition, whole-cell biocatalysis approaches have been developed that combine cell growth, extracellular production of cutinases and PET degradation into a single process [24]. However, a cost-effective extracellular production process for PET-degrading cutinase requires a high amount of the enzyme being released into the culture supernatant. In earlier studies, Su *et al*. showed a significant improvement in cell growth and especially in the extracellular production of a *T. fusca* cutinase in *E. coli* by optimising the cultivation conditions and the induction strategy [25]. Consequently, this work focused on the optimisation of the extracellular recombinant production of a leaf branch compost cutinase (LCC) [26]-based variant in *E. coli* and *C. glutamicum*. The selected cutinase variant, ICCG_DAQI_, evolved from the thermostabilized ICCG variant [27], has shown improved efficiency in the degradation of polyester fibres [10]. For expression in *C. glutamicum*, ICCG_DAQI_ was combined with different signal peptides from *B. subtilis* [20], whereas no signal peptide was used for expression in *E. coli*. Extracellular release was assessed by esterase activity using different nitrophenolic substrates to ensure comparability with other studies, and was compared between the two expression organisms. By optimising the cultivation conditions, including temperature and cultivation mode in the bioreactor, the bacterial growth of *E. coli* and thus the recombinant expression of the cutinase should be improved. Subsequently, the need for purification of extracellular cutinase was investigated by evaluating the suitability of ICCG_DAQI_-containing culture supernatants for direct hydrolysis of textile PET materials.

## Materials and methods

### Materials

Analytical grade salts, titrants and components for bacterial cell culture were obtained from Carl Roth GmbH (Karlsruhe, Germany). The casein enzymatic hydrolysate N-Z-Amine^®^ was purchased from Merck Millipore (Billerica, MA). Enzymes used for molecular cloning were from New England Biolabs (Ipswich, MA). The chromogenic substrate p-nitrophenyl acetate (pNPA) was purchased from Thermo Fisher Scientific (Waltham, MA), while p-nitrophenol butyrate (pNPB) and p-nitrophenyl palmitate (pNPP) were acquired from Sigma Aldrich (St. Louis, MO). Post-industrial PET fibres (glass transition temperature = 74.2 °C, crystallinity = 9%, specific surface area = 0.21 m^2^ g^−1^) were provided by Gneuss Kunststofftechnik (Bad Oeynhausen, Germany) [10].

### Plasmids and bacterial strains

For expression in *C. glutamicum* (DSM 20300, Leibniz Institute DSMZ-German Collection of Microorganisms and Cell Cultures GmbH) [28] the gene sequence of the cutinase variant (ICCG_DAQI_) [10] used in this work was cloned into the pEKEx2 vector using *E. coli* DH5α MCR cells [29]. The genes encoding several signal peptides (AmyE, AprE, LipA, LipB, NprE, YpjP, YwmC) originally from *B.* *subtilis* were cloned N-terminally for secretion [19, 20, 30]. For expression in *E. coli*, the pET26b-ICCG_DAQI_ construct, cloned via the NdeI and XhoI restriction sites, was transformed into BL21 (DE3) cells.

### Cultivation media

*Corynebacterium glutamicum* was cultivated in tryptic soy broth (TSB) medium (DSM 545, Leibniz Institute DSMZ-German Collection of Microorganisms and Cell Cultures GmbH), while general cultivation of *E. coli* was carried out in lysogeny broth (LB) medium (Table S1). For cultivation on plates, 15 g L^−1^ agar was added to the medium. For protein expression, either the respective medium with isopropyl-β-D-1-thiogalactopyranoside (IPTG) induction (*C. glutamicum, E. coli*) or autoinduction medium (*E. coli*) was used. For autoinduction, different types of autoinduction medium based on Studier (2005) [31] were used (Table S1).

### Cultivation in shake flasks

For seed cultures, 20 mL LB (*E. coli*) or TSB (*C. glutamicum*) medium with 50 µg mL^−1^ kanamycin was inoculated with a colony from a freshly streaked agar plate. This seed culture was incubated for approx. 14 h at 30 °C (*C. glutamicum*) or 37 °C (*E. coli*) with shaking at 180 rpm. The seed culture was used to inoculate 100 mL medium in 500 mL baffled shake flasks to an optical density at 600 nm (OD_600_) of 0.1. When protein expression was induced with IPTG in *E. coli* and *C. glutamicum*, 100 µM IPTG was added when an OD_600_ of 0.6–0.8 was reached. The cultures were grown for 24 h at 120 rpm (50 mm shaking throw, Infors-HT, Bottmingen, Switzerland) at an expression temperature of 20 °C for *E. coli* and 30 °C for *C. glutamicum*.

### Batch and fed-batch cultivation of *E. coli* in stirred-tank bioreactors

Cultivation of *E. coli* for expression of the target protein was performed in 2 L stirred-tank reactors (Getinge AB, Göteborg, Sweden) equipped with two 6-bladed Rushton turbine impellers. The basic components of LB or autoinduction medium were autoclaved together with the reactor periphery. Additives were added to the medium under sterile conditions after autoclaving (Table S1). For precultures 20 mL LB medium in a 100 mL flask was inoculated with a colony from a freshly streaked agar plate. This seed culture was incubated for approx. 14 h at 37 °C and shaken at 180 rpm and then used to inoculate 1 L of medium to an OD_600_ of 0.01. The pH was maintained constant at 6.9 by automatic addition of 2 M HCl and 2 M NaOH. Antifoam B emulsion (Sigma Aldrich, St. Louis, MO) was automatically added when foam came into contact with the foam sensor. Dissolved oxygen was maintained above a minimum of 20% saturation during the batch phase and 60% saturation during the feed phase, with the stirrer speed automatically controlled between 300 and 1000 rpm to ensure sufficient oxygen input (compressed air, 4 L min^−1^). After an initial growth phase at 37 °C, the expression temperatures were set to 20 or 30 °C. When protein expression was induced with IPTG, 100 µM IPTG was added when an OD_600_ of 0.6–0.8 (early induction) or 3 (late induction) was reached and the temperature was lowered to the corresponding expression temperature. For autoinduction, the temperature was set to the target value when an OD_600_ of 1 was reached. Samples were taken periodically during cultivation for analysis of biomass growth and sugars and determination of extracellular cutinase activity. In pulsed batch mode, sterile solutions of glycerol (20 mL, 250 g L^−1^) and lactose (20 mL, 100 g L^−1^) were added twice during the exponential growth phase, while during stationary growth lactose (20 mL, 100 g L^−1^) was added repeatedly. In fed-batch cultivations, sterile filtered lactose solution at a concentration of 100 g L^−1^ supplemented with 200 µg mL^−1^ kanamycin served as feed. A specific growth rate (μ) of 0.1 h^−1^ and yield coefficient (Y_X/S_) of 0.45 g_CDW_ g_glucose_^−1^ were used to determine the exponential feeding rate [32, 33]. Feeding started when the OD_600_ was 3, with exponential feeding sustained for 20 h. The feeding rate reached at 20 h was then maintained for further 20 h.

### Batch cultivation of *C. glutamicum* in stirred-tank bioreactors

The cultivation of *C. glutamicum* in stirred-tank bioreactors was carried out similarly to the batch mode for *E. coli*. TSB medium was autoclaved along with the reactor periphery, with additives added under sterile conditions prior to the start of cultivation. For the seed culture, 20 mL TSB medium in a 100 mL flask was inoculated with a colony and incubated at 30 °C for 24 h. The seed culture was then used to inoculate the medium in the reactor with a starting OD_600_ of 0.05. The cultivation temperature was kept constant at 30 °C. For protein expression, 100 µM IPTG was added when an OD_600_ of 0.6–0.8 was reached.

### Bis–Tris polyacrylamide gel electrophoresis

For polyacrylamide gel electrophoresis (PAGE) discontinuous Bis–Tris gels with a 4% (w/v) stacking gel and a 15% (w/v) separating gel were used [34]. Prior to use, samples were diluted in 4 × NuPAGE LDS sample buffer (Thermo Fisher Scientific, Waltham, MA) and denatured at 95 °C for 15 min. Gel electrophoresis was performed under reducing, denaturing conditions at 35 mA per gel using 2-(N-morpholino)ethanesulfonic acid (MES) buffer (1x) supplemented with 500 µL 1 M sodium bisulfite as reducing agent. Protein size was determined using the Precision Plus Protein Standard Dual Colour (Bio-Rad, Hercules, CA). For Coomassie Blue staining the gels were microwaved with Coomassie R250 (0.04% (w/v), 10% methanol, 10% acetic acid) and incubated 15 min at room temperature. Destaining was done overnight for approx. 16 h with 10% acetic acid. Gels were documented using a gel documentation system (Bio-Rad, Hercules, CA). The molecular weight of the target protein was calculated using ImageJ and compared with the theoretical molecular weight obtained from ProtParam (ExPASy) [35, 36]. Densitometric analysis using ImageJ was used to determine protein purity [35].

### Optical density and cell dry weight

To determine the OD_600_, the bacterial suspension was diluted in triplicate with 0.9% (w/v) NaCl to match the linear range of the photometer and transferred to 10 mm polystyrene cuvettes. The absorbance was then measured using a photometer (Implen, Munich, Germany) at a wavelength of 600 nm.

As various factors can affect the measured optical density when cultivating *E. coli*, especially when expressing recombinant proteins, the cell dry weight (CDW) was also determined. CDW was determined gravimetrically by drying 2 mL reaction tubes (Eppendorf) at 60 °C for at least 24 h. The preweighed reaction tubes were filled with 2 mL cell suspension and centrifuged at 10,000 g for 10 min. The supernatant was discarded and the cell pellets were dried at 60 °C for at least 24 h. The tubes were weighed again and the CDW in g L^−1^ was calculated from the difference in weight between the initial and final values.

### Colony forming units and plasmid stability

For the determination of colony forming units (CFU), samples were diluted with sterile 0.9% (w/v) NaCl. 100 µL were then plated on agar plates and incubated overnight at the appropriate temperature. The number of colonies per plate was counted and the CFU per mL calculated.

To analyse plasmid stability, samples were diluted to obtain between 10 and 200 colonies per plate and 100 µL were plated on agar plates with kanamycin (50 µg mL^−1^) and without. After overnight incubation, the colonies were counted and the fraction of plasmid-bearing cells was determined from the ratio of CFU on the kanamycin and non-kanamycin containing plates.

### Membrane integrity assay

The determination of membrane integrity using the fluorescent dyes 4′,6-diamidin-2-phenylindol (DAPI) and propidium iodide (PI) is described in detail in the supplementary information.

### Chromatographic analysis of sugars

High performance liquid chromatography (HPLC) measurements were performed using an Aminex HPX-87H column (Bio-Rad, Hercules, CA) in a Shimadzu HPLC system (Kyoto, Japan). The flow rate was set to 0.6 mL min^−1^ at 65 °C using 5 mM H_2_SO_4_ as mobile phase. 20 µL of homogenized and filtered (PTFE) samples were injected for analysis. Identification and quantification of glucose, lactose and glycerol by a refractive index detector (RID) were performed by corresponding standard curves. The retention times for lactose, glucose and glycerol were 7.5 min, 8.9 min, and 13.1 min, respectively.

### Cutinase activity assay

Various nitrophenol substrates—pNPA, pNPB and pNPP—were used for the spectrophotometric determination of the extracellular esterase activity to ensure comparability with the literature where different substrates are commonly reported. Substrate stock solutions of pNPA, pNPB and pNPP were prepared in acetonitrile (pNPA) or isopropanol (pNPB and pNPP), respectively. The appropriately diluted culture supernatant was pre-incubated in the required volume of the suitable buffer in a 96-well microtiter plate at 37 °C according to the assay procedures already described in the literature [17, 19, 37]. Depending on the substrate, either 20 mM Tris, 10 mM NaCl, pH 8 (pNPA, pNPB) or 50 mM phosphate buffer, pH 8 containing sodium desoxycholate and gum arabic (pNPP) was used as reaction buffer [17, 19, 37]. After addition of the substrate, the release of p-nitrophenol was monitored immediately by measuring the absorbance at 405 nm for 10 min at 37 °C in a plate reader (Infinite M Nano + , Tecan Group AG, Männedorf, Switzerland) [37]. The absorbance of a blank (non-enzymatic sample), to which only the assay buffer was added instead of the supernatant, was also measured and subtracted when calculating the activities of the supernatant-containing samples. For each activity measurement, a control was also analysed using the supernatant from the cultivation of cells containing the empty vector pEKEx2 (*C. glutamicum*) or pET26b (*E. coli*). The p-nitrophenol concentration could be quantified using the p-nitrophenol extinction coefficient (17.4 mM^−1^ cm^−1^) determined under reaction conditions [10]. Finally, the linear slope obtained was used to calculate the esterase activity per mL of culture supernatant, where one unit of esterase activity was defined as the release of 1 μmol p-nitrophenol per minute.

### Protein purification

The protein of interest was purified from either extracellular or intracellular sources. After harvesting (4500 g, 15 min, 4 °C), the cell pellet was resuspended in immobilized metal affinity chromatography (IMAC) binding buffer (20 mM Tris, 300 mM NaCl, 10 mM imidazole, pH 8) or the corresponding culture supernatant was used. For intracellular protein isolation, cells were first disrupted on ice using ultrasound (Bandelin MS 73, Berlin, Germany; 60% amplitude, 3 × 5 min). After a heat shock at 60 °C for 20 min, the cell lysate centrifuged (15,000 g, 45 min, 4 °C) to pellet the cell debris at, followed by filtration (0.45 μm). In contrast, the cell culture supernatant was pre-treated only by heat shock (60 °C, 20 min) and filtration (0.45 μm). In both cases, the filtrates were applied to an equilibrated HisTrap FF crude column (Cytiva, Chicago, IL). Unbound proteins were washed out with binding buffer over 10 column volumes (CV) and the target protein was then eluted with elution buffer (20 mM Tris, 300 mM NaCl, 500 mM imidazole, pH 8) over 5 CV. After elution, the protein solution was rebuffered in storage buffer (20 mM Tris, 300 mM NaCl, pH 8) through PD-10 desalting columns (Cytiva, Chicago, IL). The resulting purified protein solution was aliquoted and stored at − 80 °C until further use. Protein purity was determined by PAGE with Coomassie staining, while quantitative determination of protein concentration was performed using a Pierce BCA Protein Assay Kit (Thermo Fisher Scientific, Waltham, MA) according to the manufacturer’s instructions.

### PET hydrolysis

A Labfors 3 system (Infors HT, Bottmingen, Switzerland) with a working volume of 1 L and a 6-bladed Rushton turbine impeller was used for PET hydrolysis. The temperature of the buffer was maintained at 70 °C by an internal heating element and an external circulator thermostat (Julabo, Seelbach, Germany). Hydrolysis studies were performed in 100 mM potassium phosphate buffer at pH 9 using 10 g L^−1^ PET fibres and stirring of 300 rpm. Enzymatic hydrolysis was initiated by the addition of either IMAC purified enzyme or unpurified cultivation supernatant. pH control was achieved by the automatic addition of 1 M NaOH. Samples were taken periodically through the sampling port and stored at − 20 °C until the analysis of the hydrolysis products terephthalic acid, ethylene glycol, mono-(2-hydroxyethyl)terephthalic acid (MHET) and bis-(2-hydroxyethyl)terephthalic acid (BHET) by HPLC as previously described [10].

### Statistical analysis

A two-sided t-test for independent samples (Excel) was used to determine whether the differences between individual results were statistically significant.

## Results

### Comparison of expression hosts

**Fig. 1:**
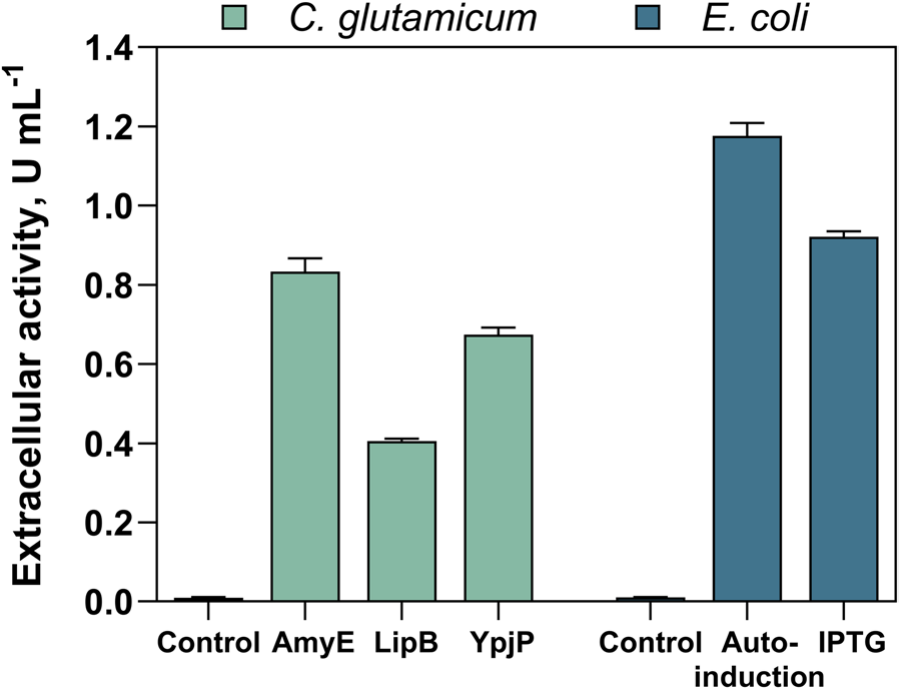
Extracellular activities determined by enzymatic hydrolysis of pNPB using *E. coli* and *C. glutamicum* cultures in stirred-tank reactors after 24 h. Different signal peptides were used for the secretion of the target protein in *C. glutamicum* cultures. For *E. coli*, induction by autoinduction and IPTG was compared. As controls, *C. glutamicum* was cultivated with only the vector pEKEx2 and *E. coli* with the vector pET26b. Each bar represents the mean ± standard deviation of three hydrolysis measurements

In this study, the secretion efficiency of the LCC variant ICCG_DAQI_ [10] in the host organism *C. glutamicum* was evaluated using seven signal peptides originally obtained from *B. subtilis* (AmyE, AprE, LipA, LipB, NprE, YpjP and YwmC). These peptides have been shown in previous research to be effective in enhancing cutinase secretion in *C. glutamicum* [20, 30]. When the signal peptides were combined with the cutinase variant ICCG_DAQI_, all peptides consistently resulted in substantial extracellular activity, confirming successful secretion of the target protein into the culture medium. However, different levels of extracellular activity could be assessed, which suggests a diverse suitability of the different signal peptides for cutinase secretion as shown before [30]. Notably, the signal peptides AmyE, LipB, and YpjP showed superior extracellular activities in preliminary tests in baffled shake flasks (Figure S1). These three signal peptides were then further used for the expression in *C. glutamicum* in stirred-tank reactors and compared with the extracellular release when using *E. coli* (Fig. 1**)**.

In the stirred-tank reactor maximal secretion results (0.8 ± 0.01 U mL^−1^) were seen for *C. glutamicum* cultivation with the signal peptide AmyE. We could also measure an extracellular cutinase activity for *E. coli* cultures in stirred-tank reactors, regardless of whether protein expression was induced by the induction agent IPTG or by autoinduction. Using IPTG induction, the activity observed was similar to that of *C. glutamicum* with the signal peptide AmyE. When cutinase production was induced by autoinduction, the overall maximum activity of 1.2 ± 0.1 U mL^−1^ was measurable, which was significantly (p ≤ 0.01) higher than the best results obtained with *C. glutamicum*. When we compared the extracellular activity versus the amount of substrate used, the highest yields were also achieved for *E. coli* using the auto-induction medium (Figure S2). When cells containing the empty vector were cultivated under the same conditions and therefore no cutinase was expressed, the measurable extracellular activity of the control was ≤ 0.01 U mL^−1^ (Fig. 1).

### Characterisation of *E. coli* membrane integrity

The release of cutinase by *E. coli*, in contrast to the expected intracellular accumulation under these conditions, suggested a destabilization of membrane integrity, as targeted release via a secretory pathway was estimated to be very unlikely or even not possible [38]. To study this process in more detail, several tests on membrane integrity were carried out. On the one hand, the integrity of a cell can be determined by its vitality and thus its ability to divide when determining CFU. Compared to the control culture (no induction), the number of CFU with autoinduced protein expression was reduced to 28%. On the other hand, a membrane integrity assay was also used for further analysis using DNA-intercalating fluorescent dyes, specifically PI and DAPI. While DAPI is membrane-permeable, PI can only penetrate a perforated membrane, but not intact cell membranes. The combined use of both dyes allowed an assessment of the amount of cells with intact membranes [39, 40]. By calibrating different ratios of intact to non-intact cells, we could determine a relative membrane integrity value for the autoinduced *E. coli* culture of 32.3 ± 11.7%, and 96.6 ± 32.3% for the control (*E. coli* pET26b-ICCG_DAQI_ in LB medium without induction) (Figure S3). Even though the standard deviations of the respective values were relatively high, there was a significant difference between the membrane integrity values of the induced and non-induced culture (p ≤ 0.05). Therefore, although accurate determination of membrane integrity is challenging, the combined evidence from CFU and the fluorescence assay strongly suggested a substantial amount of induced cells showed impaired membranes, facilitating the release of the target protein.

### Optimisation of cultivation conditions in batch and fed-batch stirred-tank reactors

Without prior optimisation for extracellular expression, we observed an increased release of the cutinase ICCG_DAQI_ in the culture supernatant with *E. coli* compared to *C. glutamicum*. Because of these promising initial results, the cell densities and corresponding recombinant cutinase titres should be further improved for *E. coli.* Consequently, the following sections will focus on the specific aspects that have been identified for further analysis and modification: medium composition, induction mechanism, expression temperature and carbon source.

### Medium composition

To improve bacterial growth and the cutinase production, the autoinduction medium used for preliminary cultivation was supplemented with magnesium, ammonium, sulphur, free amino acids, lactose and trace elements (Autoinduction_supplemented_) (Table 1). This resulted in a better growth (OD_600_ = 18.5, CDW = 6.0 g L^−1^), corresponding to an increase of 52% and 60% respectively compared to preliminary tests. A similar trend was generally observed in the OD_600_ and CDW measurements. After 48 h, the final extracellular activity was 37.8 ± 2.8 U mL^−1^ (Table 1). Focusing only on the first 24 h of cultivation, as in Fig. 1, the extracellular activity was comparatively increased by a factor of 5. However, if we analysed the extended cultivation period of 48 h, the measured maximum showed an absolute increase by a factor of 32 compared to the preliminary experiments.

**Table 1:** Comparison of OD_600_, CDW and extracellular activity (pNPB assay) in the main *E. coli* cultivations of this work in stirred-tank bioreactors at different temperatures and cultivation modes after 48 h

Cultivation conditions			OD_600_, −	CDW, g L^−1^	Extracellular activity, U mL^−1^	μ_max_, h^−1^	Y_CDW_, kU g_CDW_^−1^	Y_Substrate_, kU g_Substrate_^−1^
Mode	Medium	Temperature						
Batch	LB_basic_ + IPTG	20 °C	10.0 ± 0.3	3.3 ± 0.3	2.4 ± 0.03	–	0.7	0.24
Batch	Autoinduction_basic_	20 °C	12.2 ± 0.7	3.8 ± 0.04	7.6 ± 0.2	–	2.0	1.0
Batch	LB_supplemented_ + IPTG	20 °C	15.5 ± 0.1	4.9 ± 0.5	11.9 ± 1.6	0.27	2.4	1.2
Batch	Autoinduction_supplemented_	20 °C	18.5 ± 0.1	6.0 ± 0.3	37.8 ± 2.8	0.27	6.3	3.3
Batch	Autoinduction_supplemented_	30 °C	16.6 ± 0.1	6.5 ± 0.6	84.7 ± 2.7	0.58	13.0	7.4
Pulsed batch	Autoinduction_supplemented_	30 °C	23.0 ± 0.1	8.0 ± 0.1	118.7 ± 2.4	0.55	14.8	5.5
Fed-batch	Autoinduction_supplemented_	30 °C	26.5 ± 0.1	10.9 ± 0.3	136.6 ± 8.2	–	12.6	2.7

Also shown are the maximum growth rates (µ_max_) during the exponential phase, and the yield Y of extracellular activity in terms of CDW and substrate consumption. Means and standard deviations of three technical replicate measurements are shown. Values not determined are indicated by ‘–'

### Induction mechanism

The casein hydrolysate N-Z-Amine^®^, magnesium sulphate, trace elements and 10 g L^−1^ glucose as a carbon source were added to the LB medium (LB_supplemented_) to test whether these additives also had a positive effect on IPTG induction as in the supplemented autoinduction medium. Even though a substantial improvement in extracellular activity was observed compared to the initial results using the basic LB medium, this resulted in lower measurable extracellular activities compared to the results obtained with autoinduction medium (Table 1). PAGE analysis also showed reduced accumulation of extracellular proteins compared to the autoinduction approach (Figure S4 A). We also found that induction at a higher cell density (OD_600_ = 3, IPTG concentration = 500 µM), intended to enhance biomass before inducing expression, did not yield in a higher extracellular protein release compared autoinduction cultures (Table S2). As autoinduction medium uses a higher concentration of kanamycin due to increased phosphate salt concentrations, it was necessary to test whether this had an effect on plasmid stability and consequently on protein expression. However, when comparing cultures with different kanamycin concentrations (50 and 200 µg mL^−1^) under otherwise identical conditions, no substantial difference in plasmid stability was observed (Table S2).

### Cultivation temperature

**Fig. 2:**
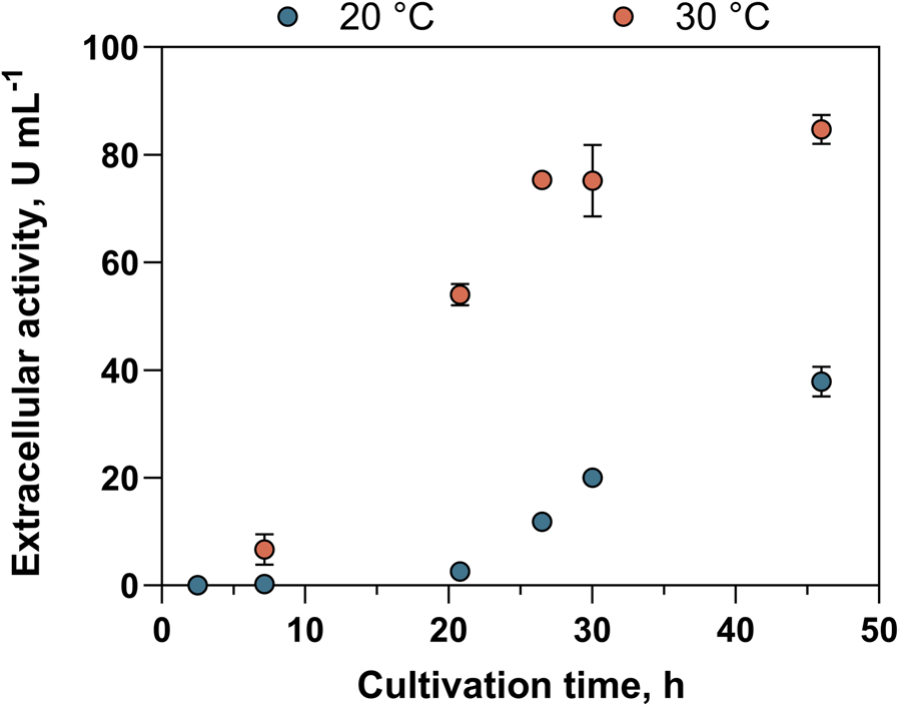
Extracellular activities determined by enzymatic hydrolysis of pNPB. Protein expression in *E. coli* using autoinduction media (Autoinduction_supplemented_) in stirred-tank reactors was monitored at 20 °C (blue) and 30 °C (red) expression temperature. Error bars represent the standard deviation (n_technical_ = 3), although they may be too small to be visible

For the target cutinase of this work, a low expression temperature—usually between 16 and 21 °C —is widely favoured in the literature [27, 41–43]. Therefore, in this work we analysed the extracellular accumulation of the cutinase ICCG_DAQI_ at the usual expression temperature of around 20 °C as well as at 30 °C. Figure 2 shows the corresponding extracellular activities of the two bioreactor cultivations with total substrate consumption after 48 h for both cultivations.

For the *E. coli* culture 30 °C the maximum growth rate was found to be twofold higher compared to growth at 20 °C (Table 1). Similarly, the maximum cutinase release rate was significantly (p ≤ 0.01) increased by 1.9-fold at 30 °C, reaching 3.5 ± 0.1 U mL^−1^ h^−1^ compared with 1.9 ± 0.2 U mL^−1^ h^−1^ at 20 °C. This acceleration, together with the earlier start of cutinase release, resulted in a 2.3-fold increase in absolute extracellular activity values at the end of cultivation (84.7 ± 2.7 U mL^−1^ for 30 °C vs. 37.8 ± 2.8 U mL^−1^ for 20 °C), with similar substrate consumption observed in both conditions (Fig. 2, Table 1). The final yields related to the biomass were 6.7 U mg_CDW_^−1^ and 13.0 U mg_CDW_^−1^, respectively, for expression temperatures of 20 °C and 30 °C. An earlier release after the start of cultivation and a stronger extracellular accumulation of the protein of interest was also evident from the bands corresponding to the protein of interest on PAGE (Figure S4 B and C).

### Carbon source feeding

**Fig. 3:**
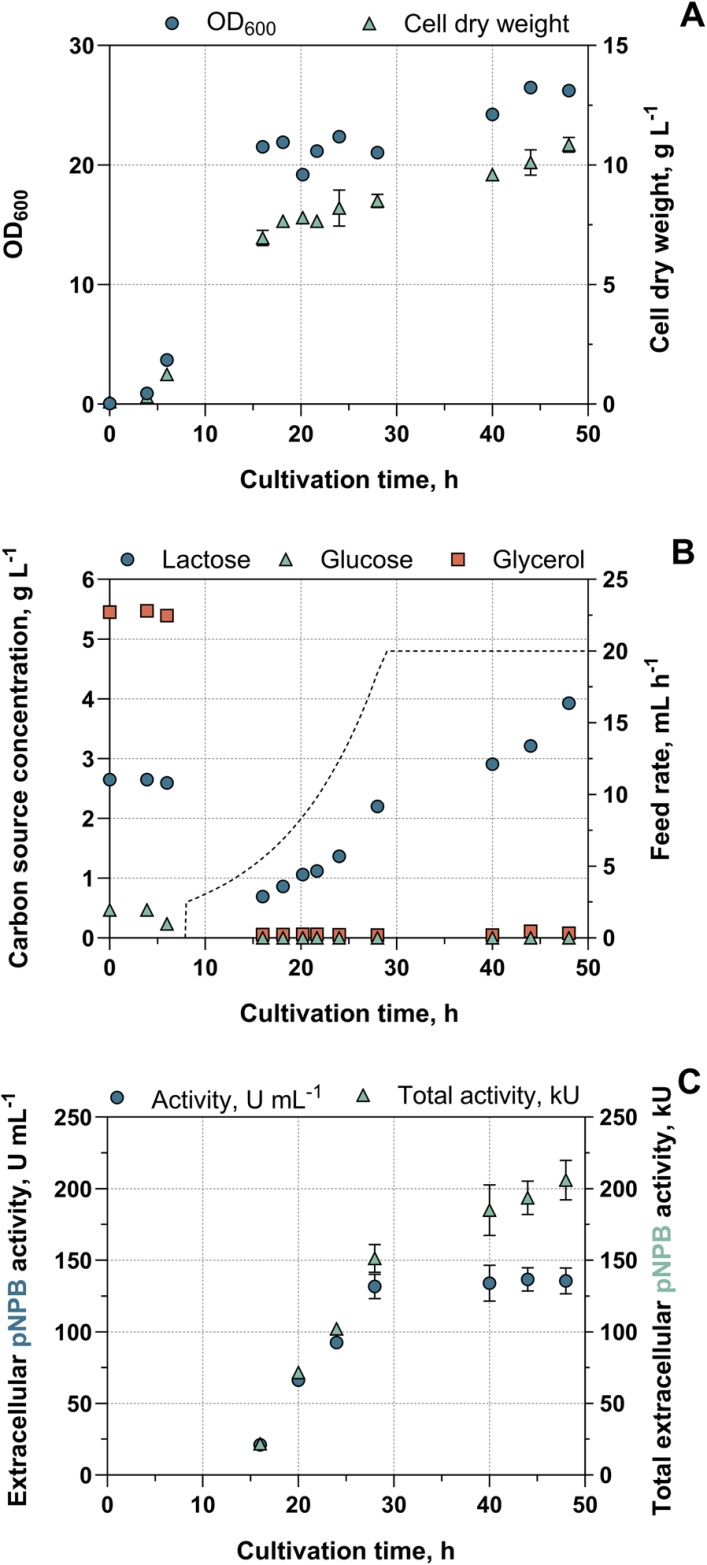
Fed-batch production of ICCG_DAQI_ using *E. coli* using autoinduction media (Autoinduction_supplemented_) in a stirred-tank reactor at an expression temperature of 30 °C and lactose feeding. OD_600_ and CDW (**A**) as well as carbon source concentrations from HPLC analysis and dashed feed profile (**B**) are shown over the cultivation time. In addition, extracellular esterase activity is shown in U mL^−1^ and total extracellular esterase activity in kU, taking into account the increasing volume of medium from the fed-batch approach, using pNPB as substrate for both measurements (**C**). Error bars represent the standard deviation are provided for OD_600_, CDW and extracellular activity (n_technical_ = 3), although they may be too small to be visible

**Fig. 4:**
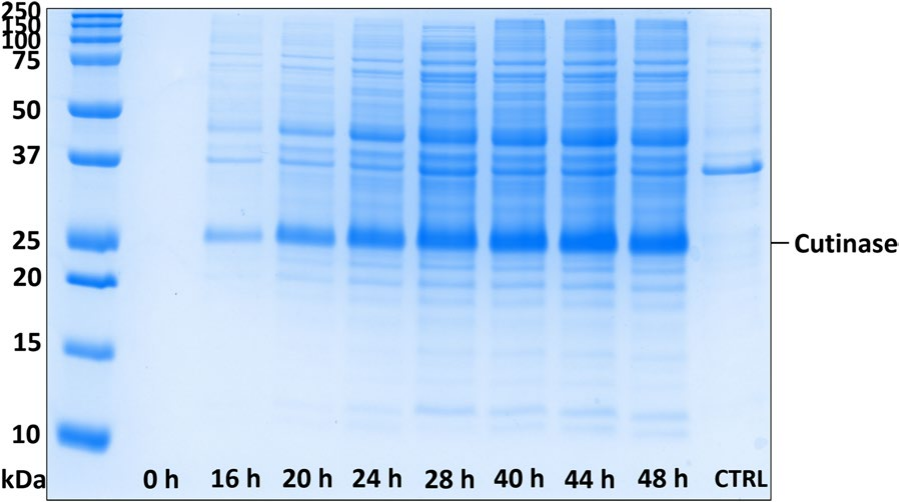
PAGE analysis of fed-batch *E. coli* cultivation supernatants (1:4 diluted) over time when using autoinduction and lactose as a feed. The temperature for protein expression was set to 30 °C. The bands with a calculated molecular weight of about 28.3 kDa [35] correspond to the protein of interest with a theoretical molecular weight of 28.9 kDa [36]. As a control (CTRL) a sample (1:4 diluted, 48 h) from a fed-batch *E. coli* cultivation with the empty vector was used

Batch cultivation at 30 °C yielded a biomass of 6.5 g L^−1^, with a noticeable stagnation in growth after the first 10 h of exponential growth (Figure S5 A). As this was temporally related to the strongly decreasing concentrations of the carbon sources glucose, lactose and glycerol, in a further batch cultivation we analysed the pulsed addition of the carbon sources glycerol and lactose, with lactose being also the inducing agent (Figure S6). With this improved carbon source supply by periodic manual addition of glycerol and lactose, a significantly higher OD_600_, CDW and extracellular activity of 118.7 ± 2.4 U mL^−1^ were achieved after 48 h of cultivation (Table 1, Figure S6). This corresponds to an increase of 38, 44 and 40%, respectively, in comparison to a batch process without lactose pulses as described before. In view of the potential irregularities associated with pulsing, we incorporated a substrate feed. Fed-batch processes offer several advantages over batch processes in bioprocess engineering, particularly in terms of nutrient control, optimised resource utilisation and thus an efficient recombinant protein production. Therefore, optimal biomass production was achieved by adding lactose at an exponential feed from 8 to 28 h, followed by a constant feed rate of 20 mL h^−1^ for further 20 h (Fig. 3 B). This was found to increase OD_600_ and CDW, as well as cutinase expression and extracellular accumulation of esterase activity over the cultivation time (Table 1, Fig. 3 A and C). In particular, extracellular activity peaked at values of 5.7 ± 0.4 U mL^−1^ (pNPA assay), 136.6 ± 8.2 U mL^−1^ (pNPB assay) and 120.3 ± 7.2 U mL^−1^ (pNPP-Assay) towards the end of the cultivation period. The activity measurements with the different substrates pNPA, pNPB and pNPP correlated well in each case. However, they are not shown in the graphs for reasons of clarity, but are listed here for comparison with the literature. To include the diluting effect of the feed during fed-batch cultivation, the total activity was calculated in addition to the concentration of extracellular activity, resulting in a total activity of 206 kU at the end of cultivation. Protein accumulation data from PAGE (Fig. 4) showed a steady increase in band intensity starting at 16 h, which continued until approximately 28 h, after which the intensity reached a plateau. This correlated optically and densitometrically with the increase in activity (Fig. 3 C). From the 20 h point to the final measurement, the extracellular activity concentrations increased twofold, closely correlating with the densitometric evaluation which showed a 1.9 fold increase. Compared to the pulsed batch growth and extracellular protein production were further improved, especially considering absolute biomass and activity results, demonstrating the advantage of the lactose-fed approach.

### Purification of target protein from intra- and extracellular origin

**Fig. 5:**
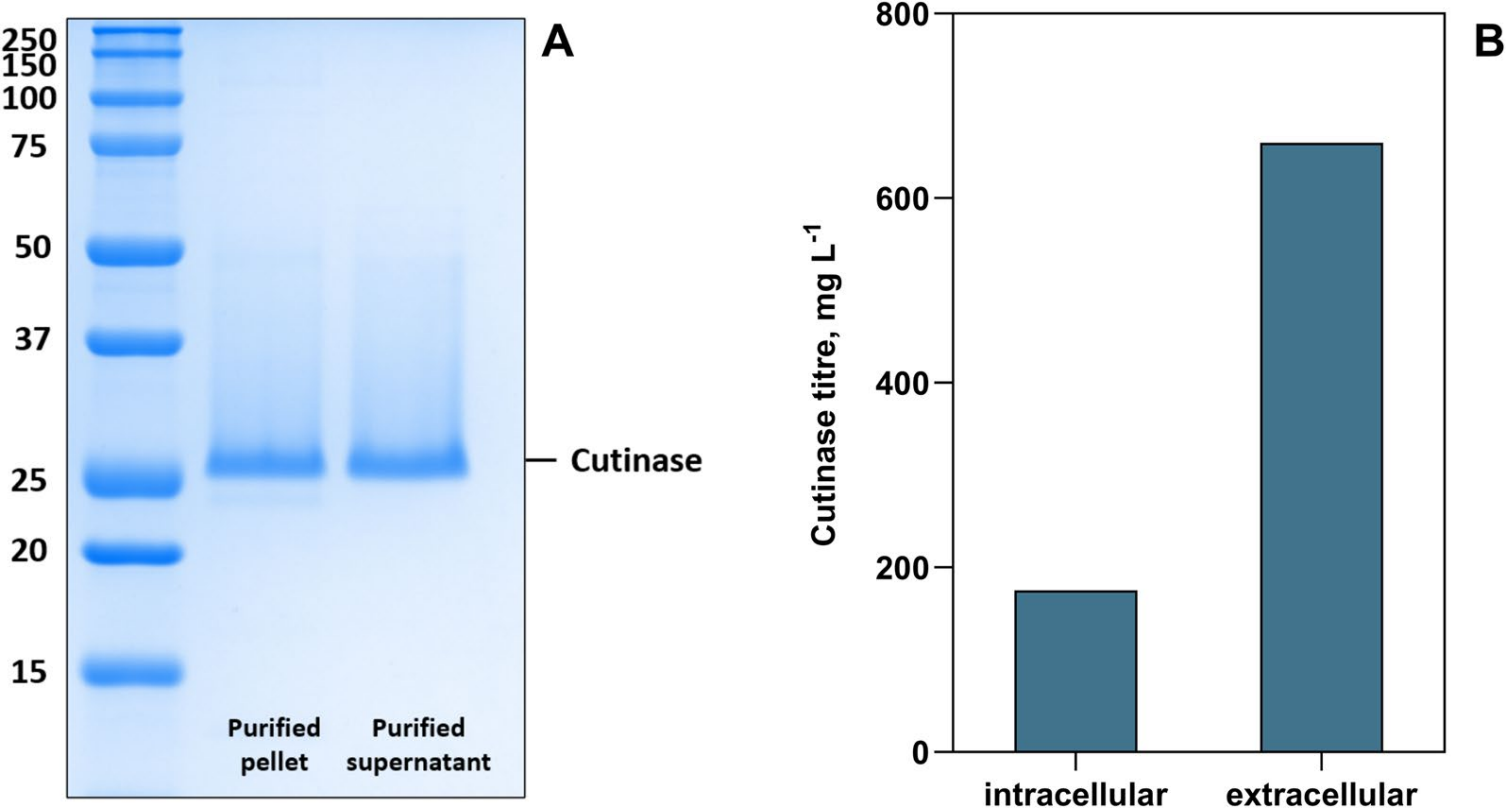
PAGE analysis of 2 µg protein from the eluate fractions from IMAC purification of pellet and supernatant after fed-batch cultivation at 30 °C (Autoinduction_supplemented_) (**A**). The bands with a calculated molecular weight of 28.4 (pellet) and 28.3 kDa (supernatant) [35] correspond to the cutinase ICCG_DAQI_ with a theoretical molecular weight of 28.9 kDa [36]. **B** Titre of soluble cutinase ICCG_DAQI_ from intracellular (pellet) and extracellular (supernatant) origin (n = 1). Intracellular cutinase and total protein concentrations were determined after disruption of a cell pellet corresponding to an equivalent volume of culture supernatant

The results of this work so far demonstrated that during the expression of recombinant cutinase in *E. coli*, a substantial amount of the cutinase ICCG_DAQI_ was found in the extracellular space, i.e., in the cultivation supernatant. Also, the PAGE analysis clearly showed an excess of the target protein in the cultivation supernatant compared to the intracellular space (Figure S7). Because cell disruption can lead to protein losses, due to incomplete disruption or protein damage by heat or cavitation, we compared the protein yields from intra- and extracellular origin to analyse the optimal source for protein isolation [44, 45]. After 48 h of fed-batch autoinduced expression at 30 °C, the culture was centrifuged, resulting in a cell pellet and supernatant fraction. While the culture supernatant was only treated by heat shock at 60 °C, the pellet had to be resuspended, disrupted by sonication in repeated cycles, heat shocked, centrifuged and filtered before being applied to the IMAC column. After a washing step, the target cutinase was eluted with imidazole under the same conditions. The chromatograms (Figure S8) clearly showed a substantial increase in the UV signal peak by a factor of 3 when the supernatant sample was eluted compared to the protein elution from the pellet sample. This observation aligns well with the calculated titre of 660 mg L^−1^ for the cutinase ICCG_DAQI_ in the supernatant and 175 mg L^−1^ for ICCG_DAQI_ in the equivalent amount of the disrupted cell pellet, corresponding to a factor of 3.8 (Fig. 5). Thus, approximately 80% of the total amount of the cutinase was found extracellularly. With a total protein titre of 5.7 g L^−1^ in the supernatant, the relative amount of target cutinase was 11.6%. In the disrupted cell pellet, 7.0% of the total soluble protein was the target cutinase ICCG_DAQI_. A further increase towards complete release could be achieved by thermal or chemical treatment, as incubation at 60 °C could increase the extracellular cutinase activity further. After 20 min the extracellular activity was increased by 16 U mL^−1^ to 154 ± 3.5 U mL^−1^ (Figure S9). Such thermolysis approaches for *E. coli* were also found to be efficient for thermostable enzymes in the literature [46]. The PAGE analysis showed a purification result from the supernatant with a purity of 96.6%, which is comparable to the results obtained with the established protocol for the purification of the target protein from intracellular origin (Fig. 5).

### Verification of hydrolytic activity in PET degradation

**Fig. 6:**
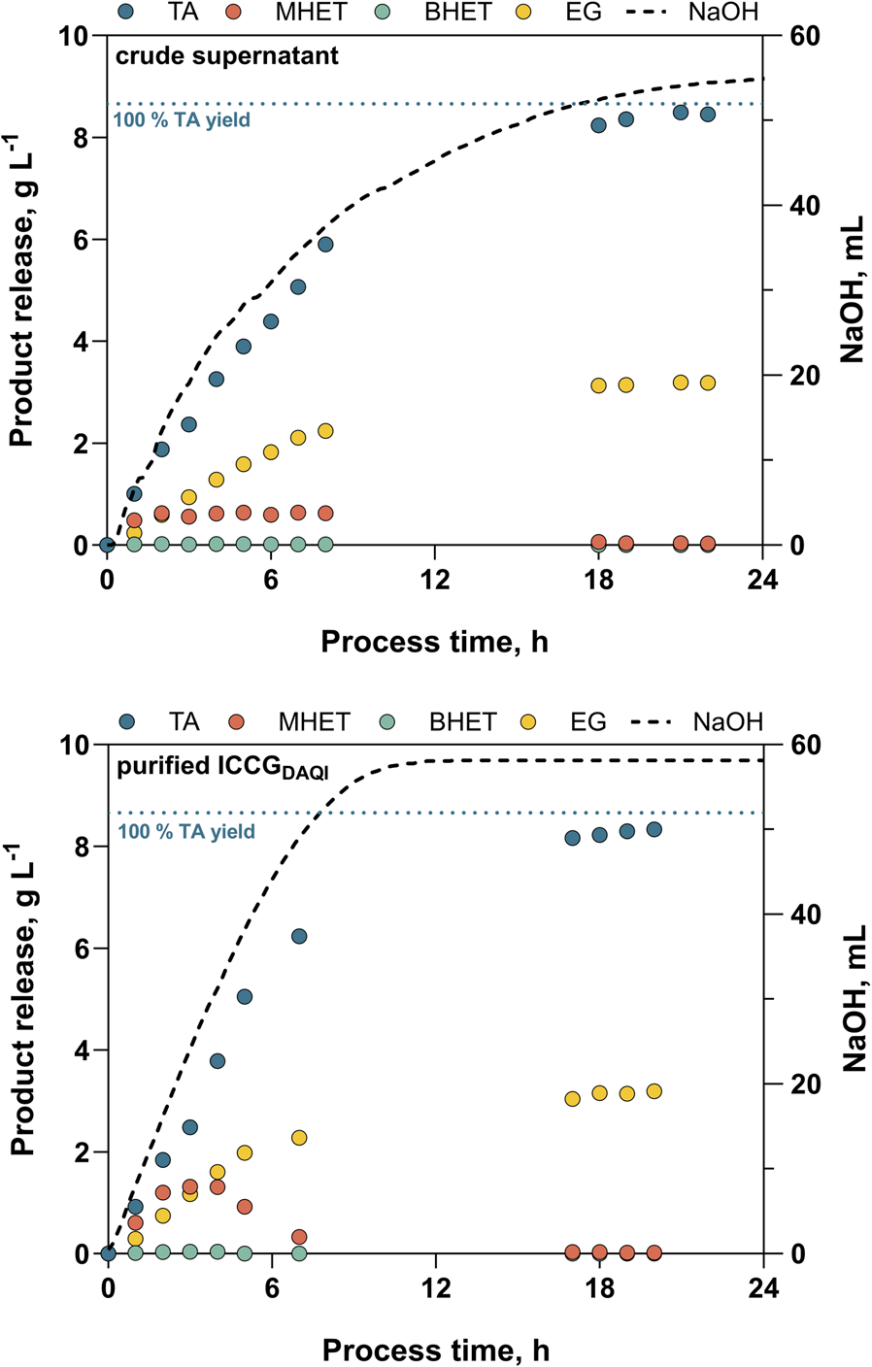
Release of terephthalic acid (TA), MHET, BHET and ethylene glycol (EG) during hydrolysis using either 5% crude *E. coli* supernatant (corresponds to 3 mg_ICCGDAQI_ g_PET_^−1^) or IMAC-purified ICCG_DAQI_ (1 mg g_PET_^−1^). NaOH was automatically added to keep the pH constant at a value of 9, whose curve (dashed line) also corresponds to PET hydrolysis. The terephthalic acid concentration corresponding to a yield of 100% is indicated by the blue dashed line

To avoid the purification of the protein altogether and thus achieve a much more cost-effective hydrolysis, the impact of using untreated culture supernatant on hydrolysis was investigated (Fig. 6). In addition to the IMAC-purified culture supernatant (see previous chapter), 5% untreated culture supernatant was used to hydrolyse 10 g of polyester fibres at 70 °C on the 1-L scale.

Compared to the use of purified protein, a slightly slower hydrolysis of the solid substrate was observed. The product release, shown by HPLC measurements and the corresponding NaOH addition curve, stagnated after 18 h compared to 12 h for the purified enzyme. Notably, the culture supernatant used in this experiment contained 3 mg of the cutinase ICCG_DAQI_ per gram_PET_, yet the hydrolysis rate was lower than that observed with 1 mg_purified cutinase_ g_PET_^−1^. The presence of other components in the culture supernatant might have contributed to the reduced efficiency of the enzyme. Nevertheless, both hydrolysis approaches showed almost complete PET conversion. In the case of the crude supernatant, this resulted in 97.8% and 98.6% yields of the hydrolysis products terephthalic acid and ethylene glycol after 20 h. For the purified protein, the yields were 96.4% and 98.8% for terephthalic acid and ethylene glycol, respectively. However, with respect to the effective enzymatic activity used, the crude supernatant performed well highlighting options for purification free application. The hydrolysis main product terephthalic acid could be effectively precipitated and recovered by acidification. At pH values below 4, recoveries of over 95% were possible. At a pH of 2.5, which is often used in the literature [47], recovery rates of up to 99.2% were possible in this work. Nuclear magnetic resonance (NMR) spectroscopic analysis of the recovered terephthalic acid showed that neither the hydrolysis products using the purified enzyme nor the unpurified culture supernatant contained impurities other than minor traces of water, compared to commercial terephthalic acid of > 99% purity (Figure S10).

## Discussion

In the literature, *C. glutamicum* is described as an efficient secretory organism that can express different cutinases extracellularly using various signal peptides [30, 48, 49]. For the ICCG variant, Helleckes et al. showed an extracellular activity of 0.8 U mL^−1^ (pNPP) for *C. glutamicum* using the signal peptide LipB on the 1-L bioreactor scale [19]. This activity is in the same range as in this work, where AmyE as the best signal peptide showed an activity of 0.8 U mL^−1^ (pNPP assay), whereas LipB showed a lower activity of 0.2 U mL^−1^ in our study. Differences in these values may be due to variations in media composition, such as minimal versus full medium, affecting chaperone synthesis, differences in cultivation conditions and slight modifications of the enzyme variant used in our study. The use of alternative vectors with tighter control may improve protein production, as the observed reduction in yield may be linked to leaky expression of the pEKEx2 vector [50]. However, as we observed extracellular expression of the cutinase ICCG_DAQI_ not only in *C. glutamicum*, but also in *E. coli*, extracellular cutinase formation is not restricted to secretory organisms, but also occurs in *E. coli* even without a signal peptide. This effect might be due to the expressed protein itself. As a gram-negative bacterium, *E. coli* has a three-layered cell wall consisting of an inner or cytoplasmic membrane, a peptidoglycan layer and an outer membrane [51]. The peptidoglycan layer of *E. coli* is remarkably thin, measuring less than 10 nm, whereas gram-positive bacteria have a much thicker, multi-layered peptidoglycan layer of 30 to 100 nm. The inner and outer membranes consist of (phospho)lipid layers, each no more than 7 nm thick [51, 52]. While the peptidoglycan layer allows the permeation of molecules below 50 kDa, the outer membrane acts as an efficient selective permeation barrier [53, 54]. The protein of interest, a catalytically highly active cutinase, is an ester hydrolysing enzyme with the theoretical ability to hydrolyse ester bonds within the lipid bilayer, disrupting its integrity. Phospholipase activity has been demonstrated in the past for some cutinases, e.g. from *Thermobifida fusca* [23]. If the induction process leads to a gradual intracellular accumulation of cutinase, it is conceivable that the ester bonds could be affected, leading to permeabilisation of the cellular lipid bilayer. As a result of this damage to the primary protective barrier of *E. coli*, the natural existing permeability of the thin peptidoglycan layer in *E. coli* can lead to the release of the intracellularly accumulated 29 kDa protein, which would provide a plausible mechanism for the observed membrane damage and subsequent release of the target protein. Evidence for this assumption can also be found in the literature. When a cutinase from *T.* *fusca* was expressed in *E. coli* BL21 (DE3), significantly higher permeabilities of the inner and outer membranes were found [23, 25]. However, β-galactosidase as a reference protein with a high molecular weight of 467 kDa (tetramer) [55], was released to a substantially lower extent than the cutinase, so that a complete lysis of the cell was not assumed [23]. In contrast, when a genetically inactivated cutinase variant was expressed, no membrane damage occurred, resulting in no release into the medium [23, 25]. When *E. coli* with the empty vector was cultured in shake flasks, only minimal amounts of certain proteins were detected in the culture supernatant under these conditions (Figure S11). Fed-batch cultivation of *E. coli* with the empty vector in a stirred tank reactor at 30 °C resulted in a slightly increased release of these proteins into the extracellular space. Additional proteins were released in the later stages of fed-batch cultivation (> 36 h), probably as a result of cell damage due to nutrient depletion, metabolic stress and cell death (Figure S12). However, the extent of protein release observed under both cultivation conditions was significantly lower than that observed during cutinase expression. This indicates that the disruption of membrane integrity when expressing the cutinase ICCG_DAQI_ is not due to mechanical stress on the cells during cultivation. Even when LCC was first described, small amounts were already reported in the culture medium of *E. coli* at 37 °C in the case of overproduction (Table 2). However, in this case the enzyme was tagged with the pelB leader sequence, which may have contributed to the release [26, 56]. In this work, we have clearly seen a substantial increase in the target cutinase in the supernatant over the duration of cultivation, but also an increase in the release of other proteins in the supernatant. Combined with the CFU results, this suggests that the membrane appears to become damaged, which seems to go beyond a selective release of the target cutinase. Instead, it is more plausible that some sort of release effect is occurring where membrane integrity is compromised to such an extent that intracellular proteins are released undifferentiated into the culture medium. Flow cytometry may be used to investigate the exact relationship between protein production and cell membrane integrity in more detailed research.

**Table 2:** Comparative analysis of extracellular activities and cutinase titres of LCC variants across different organisms

	Extracellular activity, U mL^−1^			Cutinase titre, mg L^−1^	Source
	pNPA	pNPB	pNPP		
*B.* *subtilis*	–	22.6	–	85	Oh et al*.* [17]
*C.* *glutamicum*	–	–	0.8	–	Helleckes et al. [19]
*C.* *thermocellum*	–	1.0^*^	–	–	Yan et al. [24]
*E.* *coli* BL21 (DE3)	–	–	–	8^**^	Sulaiman et al. [26]
*E.* *coli* BL21 (DE3)	–	–	–	approx. 120	Soong et al. [21]
*E.* *coli* BL21 (DE3)	5.7	136.6	120.3	660	This work
*K. phaffii*	–	–	–	200–300^**^	Shirke et al*.* [16]

–: This value was not determined in the respective study

^*^A different substrate concentration (100 µM) was used in the pNPB assay of this study

^**^Instead of the improved ICCG/ICCG_DAQI_ variants, the wild-type LCC was tested in this study

In this work, we found autoinduction to be more suitable for the extracellular expression of the cutinase variant under study than conventional induction with IPTG. Also, literature generally indicates that autoinduction outperforms classical IPTG induction, especially when dealing with toxic or challenging recombinant proteins, by reducing metabolic stress and increasing target protein yields. This advantage is due to the gradual induction process and the ability to fine-tune the timing of induction with lactose, resulting in higher cell yields and expression efficiencies [31]. In this case, metabolic stress or cell damage by the cutinase, along with the highly concentration-dependent nature of expression, which can vary depending on the target protein and organism, are possible reasons for the lower expression with IPTG [57]. Potential cell damage during IPTG induction caused by sudden high intracellular enzyme concentrations may be reduced by autoinduction, which allows a milder induction and balances enzyme production and release across a partially permeable membrane. Higher IPTG concentrations are toxic to cells, leading to reduced growth and accumulation of insoluble proteins, while low concentrations may result in insufficient expression, as observed in this case. In principle, an IPTG range between 0.05 and 2 mM is recommended, with the previously in shake flasks determined optimal IPTG concentration of 0.1 mM falling within this recommended range (Figure S13). Also, in the literature for *E. coli* and *C. glutamicum*, values in the range of 100–250 µM are reported to be optimal for the secretion of cutinases [50, 58]. However, the transition to bioreactor cultivation and changes in the culture medium may require a reassessment of the optimal IPTG induction and timing. Nevertheless, as autoinduction proved to be better in our case and is recommended in the literature as a softer induction method, no further studies were carried out with IPTG [31]. The use of the carbon source lactose as inducer is also an option for future up-scaling, as IPTG is considered too expensive for the production of low-cost recombinant proteins, especially on an industrial scale [59]. The positive effect of lactose as an inexpensive feed shown in this work is also confirmed in the relevant literature, sometimes in combination with IPTG as an additional inducer or glycerol as an alternative carbon source [60–62].

In this work in particular the media components showed a substantial influence on bacterial growth and cutinase production, as a clear increase in both parameters was observed in the supplemented version of the autoinduction medium (Table S1). This may be due to the increased concentrations of salts such as magnesium, ammonium, sulphur and trace elements, which are known to be key factors in increasing saturation cell density [31, 63]. The addition of trace elements was also found to be essential for robust growth and expression [31]. This, combined with other factors such as the potential increased availability of free amino acids from N-Z-Amine^®^ and lactose addition, presumably led to higher cutinase yields over the extended cultivation period. As shown above, also the temperature chosen for expression affected the extracellular release of cutinase, as production at 30 °C benefited from accelerated cutinase formation and increased catalytic esterase reaction rates at higher temperatures, leading to enhanced membrane permeabilisation [56, 57]. This effect may have been enhanced by the general increase in membrane fluidity at higher temperatures, facilitating membrane diffusion processes [64, 65]. Membrane integrity issues, particularly at 30 °C and during prolonged cultivation, can alter OD_600_ measurements due to reduced light scattering from compromised cells, requiring consideration of CDW [66]. Further optimisation of the feeding strategy could potentially increase activity yields relative to the carbon source used. However, in addition to the volume-based activities, the absolute activity values achieved in the fed-batch cultivation are particularly promising, with a total extracellular activity of 205 kU compared to 119 kU in the pulsed batch, demonstrating the potential of a fed-batch approach.

We have demonstrated that extracellularly accumulated cutinase can also be used to hydrolyse PET fibres. Indeed, other studies have shown that extracellularly expressed cutinases have the same properties as intracellularly expressed ones, such as temperature and pH optima and stability, as well as catalytic properties [21, 23]. Furthermore, we found that PET hydrolysis worked well under standard conditions even when only 5% unpurified culture supernatant containing 3 mg cutinase was used, indicating that purification of the expressed cutinase by IMAC may not be necessary. The slight deceleration in hydrolysis may be attributed to proteolytic activity in the unpurified culture supernatant, indicating potential protease interference with the hydrolytically active target protein and resulting in a lower effective enzyme concentration [67, 68]. The presence of culture supernatant might also affect the overall catalytic activity. Future possibilities include further separation of proteolytically active enzymes, potentially achieving hydrolysis speeds comparable to those with purified protein. Moreover, efficient use of the cutinase can be achieved by using the cultivation supernatant directly for various immobilisation approaches like magnetic nanoparticles, responsive polymers and cross-linked enzyme aggregates [69–71]. Such enzyme immobilisation strategies from the supernatant would also minimise the risk of introducing impurities or colour-altering substances from the cultivation media into the monomers utilised for circular plastic production. Using the cutinase from the culture supernatant and avoiding cell disruption, even when efficient methods such as ultrasonic disruption with four 1 min sonication cycles are available [72], offers significant advantages for extracellular protein expression. This approach not only eliminates potentially damaging steps such as heat generation and cavitation that can occur during disruption, but also reduces the need for multiple centrifugation cycles. These benefits are particularly valuable when processing larger volumes, where extracellular expression can simplify the production process [73, 74].

With a better understanding of the factors involved in expression in *E. coli*, a comparative analysis of extracellular production in different organisms is crucial to assess the overall suitability. The choice of expression system and release route ultimately depends on the specific objectives and expectations. In this study, *E. coli* with leaky release of intracellularly produced cutinase ICCG_DAQI_ showed higher titres compared to the secreting organism *C. glutamicum*. Especially for heterologous protein production secretion yields can be relatively low [75]. The faster growth rate of *E. coli* and cultivation using autoinduction medium provided additional advantages over the secretion system in *C. glutamicum* [31]. However, the membrane disintegration in *E. coli* limits continuous cultivation, when in contrast, *C. glutamicum* offers the potential for continuous production where the cutinase could be consistently harvested from the supernatant, possibly having positive effects on the space time yield. There is also potential for optimising extracellular titres in *C. glutamicum*, for example by varying the signal peptides, one of the most important factors for efficient recombinant protein secretion [20, 76]. On the other hand, the ability of *E. coli* to release proteins independently of signal peptides could be a significant advantage. While the secretion process in *C. glutamicum* is more targeted, the co-release of other proteins in *E. coli* is a potential limitation, as it may lead to interference with catalytic activity due to impurities or proteolytic degradation [75, 77]. However, this co-release could also be used as an opportunity, for example in the co-expression of other enzymes such as bacterial cellulases for use in a multi-enzyme system [78]. In conclusion, our results indicate that leaky release of intracellularly expressed cutinase was more effective in *E. coli* than secretory release in *C. glutamicum*, mainly due to the higher released cutinase titre and the ease of optimising cultivation conditions with autoinduction medium in *E. coli*. Nevertheless, the potential for continuous production and targeted secretion in *C. glutamicum* highlights its strengths for specific applications, particularly where more controlled and purer enzyme production is needed.

In addition to *E. coli* and *C. glutamicum*, also other organisms have been reported in the literature to be suitable for the extracellular production of ICCG variants (Table 2). Compared to other studies that have investigated the extracellular accumulation of ICCG variants using *E. coli*, we found a larger amount extracellularly in our work (80% compared to approximately 30%) [21]. Several variations in growth conditions may have contributed to the higher protein titres observed in our study, as we used a higher temperature during the protein production phase (30 °C) combined with a longer cultivation time. In addition, the use of lactose as both carbon source and inducer, as opposed to glucose with IPTG induction, may have contributed to the enhanced protein expression. By using autoinduction with lactose, we may have achieved a milder induction leading to more stable protein expression as described above [31, 57]. In contrast to the secreting organisms *C. glutamicum* and *B. subtilis*, our results with *E. coli* outperformed them in terms of extracellular cutinase titre and secretion efficiency as indicated by activity assays [17, 19]. In the case of *B. subtilis*, the final results of bioreactor cultivation on a 2-L scale were exceeded by a factor of 9 for the cutinase titre and 8 for the extracellular activity [17]. The space–time yield was 3.5 mg L^−1^ h^−1^ for *B. subtilis* [17] and 13.8 mg L^−1^ h^−1^ for *E. coli* (this work), although it should be noted that the *B. subtilis* results were obtained in a batch process. Further optimisation towards a fed-batch process and optimised carbon source supply would be expected to further increase biomass and protein production in *B. subtilis* [79–81]. Notably, our results also show that *E. coli* outperforms *K.* *phaffii*, known for its high yields and protein secretion capabilities, in extracellular cutinase production compared to previous literature [16, 82]. However, the lower cutinase yield observed in *K.* *phaffii* was probably due to different cultivation conditions and the use of shake flasks instead of a bioreactor environment [16]. This further highlights the significant potential and critical importance of optimising cultivation conditions to enhance extracellular cutinase production. While whole-cell approaches, such as those combining cell growth and protein expression with simultaneous hydrolysis in *C. thermocellum*, have shown highly promising results, their activities do not yet reach the levels achieved by isolated protein expression [24]. Overall, this shows that *E. coli* is able to keep up with or even outperform secreting organisms, mainly due to higher growth rates, protein expression by autoinduction and optimal nutrient supply through fed-batch cultivation [31, 83, 84]. The choice of organism and cultivation strategy regarding conditions and nutrient supply will certainly depend on the objectives to be pursued. However, it is clear that the release of cutinase into the cell culture medium opens up new possibilities for efficient downstream processing or direct use as a biocatalyst.

## Conclusion

Extracellular expression of cutinases has been demonstrated in a variety of secreting organisms. In this study, *E. coli* was found to be superior to *C. glutamicum* in both ICCG_DAQI_ titre and extracellular activity, as demonstrated by direct experimental comparison. In particular, autoinduction, an expression temperature of 30 °C and a fed-batch process for improved carbon supply were factors efficiently improving extracellular cutinase production in *E. coli*. Successful purification of the cutinase ICCG_DAQI_ from the culture supernatant was achieved, with high purities of 96.6% and final cutinase yields of 660 mg L^−1^. The use of culture supernatant with extracellular cutinase without IMAC purification enabled complete hydrolysis of post-industrial PET waste in less than 24 h, resulting in terephthalic acid yields comparable to those obtained with purified enzyme. In closing, extracellular expression in *E. coli* offers the possibility of reducing the cost of enzyme production for biotechnological recycling of PET products. Such new opportunities for optimising time, material and cost resources are crucial to addressing the growing challenge of PET waste from single-use packaging and textile products.

## Supplementary Information

Additional file 1.

## Data Availability

All data generated and analysed during this study are included in this published article and its supplementary information file. Further data is available from the corresponding author on reasonable request.
