# Supplementary material for: Comparative evaluation of the extracellular production of a polyethylene terephthalate degrading cutinase by Corynebacterium glutamicum and leaky Escherichia coli in batch and fed-batch processes
Source: Microb Cell Fact. 2024 Oct 10;23:274. doi: 10.1186/s12934-024-02547-2 (PMC11468216; doi:10.1186/s12934-024-02547-2)
Supplement: Supplementary file 1 — Additional file 1. [file 12934_2024_2547_MOESM1_ESM.pdf]

Supplementary information

Comparative evaluation of the extracellular production of a polyethylene terephthalate degrading cutinase by *Corynebacterium glutamicum* and leaky *Escherichia coli* in batch and fed-batch processes

Stefanie Fritzsche[1], Holger Hübner[1], Marco Oldiges[2,3], Kathrin Castiglione[1]

1 Institute of Bioprocess Engineering, Department of Chemical and Biological Engineering, Friedrich-Alexander-Universität Erlangen-Nürnberg, Paul-Gordan-Straße 3, 91052 Erlangen, Germany
2 Institute of Bio- and Geosciences, IBG-1: Biotechnology, Forschungszentrum Jülich GmbH, Wilhelm-Johnen-Straße, 52428 Jülich, Germany
3 Institute of Biotechnology, RWTH Aachen University, Worringerweg 3, 52074 Aachen, Germany

*Corresponding author: kathrin.castiglione@fau.de

Material and Methods

Medium composition

Table S1: Composition of the medium for cultivating *E. coli* and *C. glutamicum* in shake flasks and the bioreactor.

Medium	Basis	Additives
$Autoinduction_{basic}$	ZY (5 g L^{-1} yeast extract, 10 g L^{-1} peptone)	1x 5052 [1] 50 mM Na_2HPO_4, 50 mM KH_2PO_4, 25 mM $(NH_4)_2SO_4$, 1 mM $MgSO_4$, 200 µg mL^{-1} kanamycin
$Autoinduction_{supplemented}$	ZY (5 g L^{-1} yeast extract, 10 g L^{-1} N-Z-amine)	1x 5052 [1] 25 mM Na_2HPO_4, 25 mM KH_2PO_4, 50 mM NH_4Cl, 5 mM Na_2SO_4, 2 mM $MgSO_4$, 0.2x trace metals [1], 200 µg mL^{-1} kanamycin
LB_{basic}	10 g L^{-1} peptone, 5 g L^{-1} yeast extract, 10 g L^{-1} NaCl	50 µg mL^{-1} kanamycin
$LB_{supplemented}$	10 g L^{-1} N-Z-amine, 5 g L^{-1} yeast extract, 10 g L^{-1} NaCl	2 mM $MgSO_4$, 0.2x trace metals, 10 g L^{-1} glucose, 50 or 200 µg mL^{-1} kanamycin
TSB	17 g L^{-1} peptone (casein), 3 g L^{-1} peptone (soy), 5 g L^{-1} NaCl, 2.5 g L^{-1} K_2HPO_4	10 g L^{-1} glucose, 30 µg mL^{-1} kanamycin

Membrane integrity assay To determine membrane integrity the fluorescent dyes 4′,6-diamidin-2-phenylindol (DAPI) and propidium iodide (PI) were used. DAPI is membrane permeable and can stain cells with both intact and compromised membranes, whereas PI can only penetrate a perforated membrane but not intact cell membranes. For quantitative calibration *E. coli* containing only the pET26b vector were cultivated in baffled flasks in LB medium (10 g L^{-1} glucose, 50 µg mL^{-1} kanamycin) at 20 °C for 48 h. After harvesting, the supernatant was discarded and the cell pellet was resuspended in 0.9 % NaCl. One part of the cell suspension was diluted 1:20 in 0.9 % NaCl (100 % intact) and one part in 70 % isopropanol (100 % damaged). After 1 h incubation at room temperature, the suspensions were centrifuged (10,000 g, 10 min) and washed twice with 0.9 % NaCl. For calibration, intact cells were mixed with damaged cells to produce different proportions of cells with intact membranes (0, 20, 40, 60, 80, 100 %). Samples from 1) induced and 2) non-induced cultures (*E. coli* pET26b-$ICCG_{DAQI}$, 48 h, 20 °C; 1: autoinduction medium, 2: autoinduction medium without lactose as inducing substance) were also harvested after cultivation, resuspended in 0.9 % NaCl and further diluted. After incubation for 1 h, the suspensions were centrifuged and washed twice as described for the calibration suspensions. From each calibration mixture and the samples, 100 µL were pipetted in triplicate into black microtitre plates. 2.5 µM DAPI and 15 µM PI were added to the samples and incubated for 30 min at room temperature in the dark. The fluorescence signals were then measured in a microplate reader (355 nm/460 nm, 535 nm/617 nm). The ratio of blue (DAPI) to red (PI) fluorescence values was calculated and the percentage values for membrane integrity in the induced and non-induced samples were obtained from the resulting linear function.

NMR analysis For ^{1}H NMR spectra, 5 mg of dried sample was dissolved in 0.5 mL of DMSO-d6 (99.8 % degree of deuteration, Thermo Fisher Scientific, Waltham, MA). The measurement

was performed using an ECX-400 (JEOL, Tokio, Japan) at a frequency of 400 MHz. The spectra were analysed using the Delta NMR Data Processing Software (JEOL, Tokio, Japan).

Results

Preliminary comparison of signal peptides in *C. glutamicum*

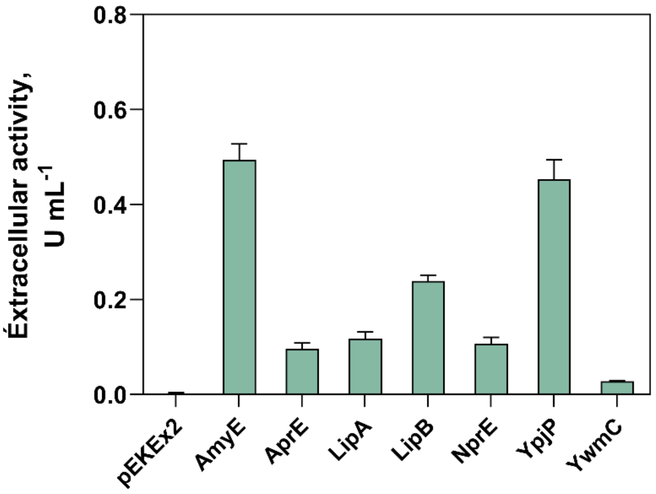

Figure S1: Extracellular activities determined by enzymatic hydrolysis of pNPB using *C. glutamicum* cultures in shaking flasks after 24 hours. Different signal peptides were used for the secretion of the target protein in *C. glutamicum* cultures. Each bar represents the mean ± SD of three hydrolysis measurements.

Extracellular activity substrate yields for *E. coli* and *C. glutamicum*

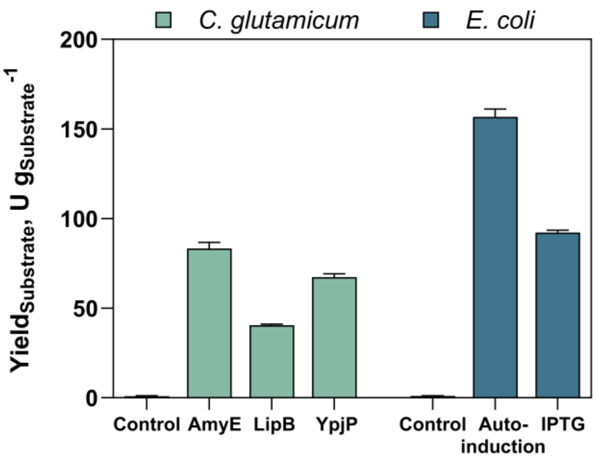

Figure S2: Yield of the extracellular activities in *E. coli* and *C. glutamicum* in relation to the amount of substrate used for the cultivation in stirred-tank reactors after 24 hours. Different signal peptides were used for the secretion of the target protein in *C. glutamicum*. For *E. coli*, induction by autoinduction and IPTG was compared. As controls, *C. glutamicum* was cultivated with only the vector pEKEx2 and *E. coli* with the vector pET26b. Each bar represents the mean ± SD of three hydrolysis measurements.

Membrane integrity

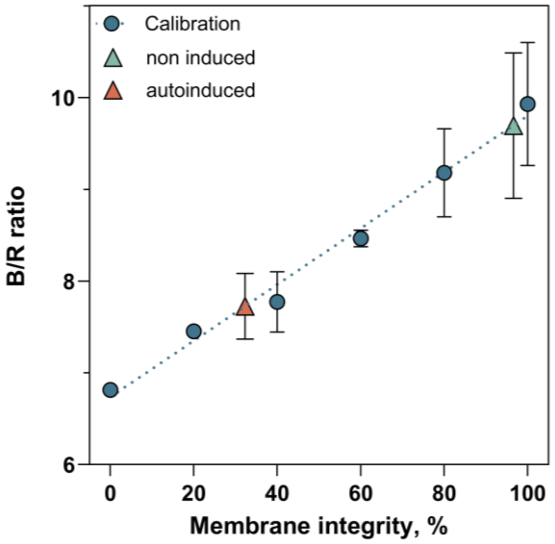

Figure S3: Ratio of DAPI (blue fluorescence, **B**) and PI (red fluorescence, **R**) measurements for cell mixtures with different dead/living cell ratios and thus membrane integrity. Samples from two *E. coli* cultures (baffled flasks, 48 h, non-induced in LB_{basic} medium (green triangle) and induced in $autoinduction_{basic}$ (red triangle)) were also stained and the B/R ratios calculated to determine the membrane integrity of the cells. The line represents a linear regression between the B/R ratio and membrane integrity for calibration (R^2 = 0.98).

Plasmid stability for different induction strategies

Table S2: Extracellular activity and plasmid stability for *E. coli* cultures with IPTG induction for different induction times and kanamycin concentrations. Values were determined after 48 hours of growth at 20 °C in $LB_{supplemented}$ in stirred tank reactors. Autoinduction values correspond to fed-batch cultivation in supplemented autoinduction medium at 30°C.

	Kanamycin concentration, µg mL^{-1}	Extracellular activity, U mL^{-1}	Plasmid stability, %	CFU, mL^{-1}
Early induction (OD_{600} = 0.6)	50	11.8	73.8	$1.3 \cdot 10^9$
	200	11.7	69.5	$1.2 \cdot 10^9$
Late induction (OD_{600} = 3)	50	10.9	-	-
Autoinduction	200	136.6	3.1	$1.3 \cdot 10^{10}$

- : This value was not determined.

Influence of temperature and induction mechanism on extracellular cutinase production

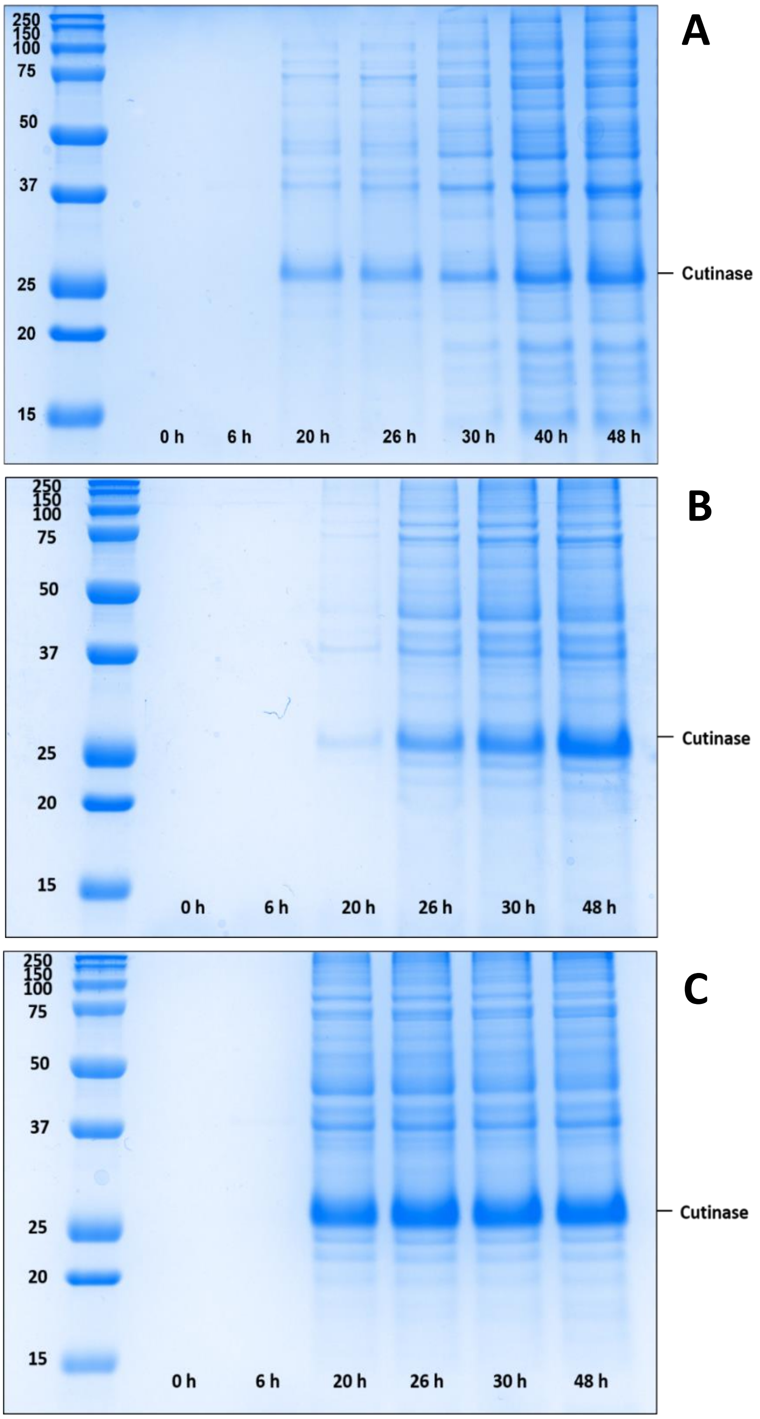

Figure S4: PAGE analysis of the extracellular proteins during *E. coli* cultivation over time. The temperature for protein expression was set to 20 °C when IPTG was used as inducer (**A**). For autoinduction the expression temperature was set to 20 °C (**B**) or 30 °C (**C**). The bands with a calculated molecular weight of about 28.3 kDa [2] correspond to the protein of interest with a theoretical molecular weight of 28.9 kDa [3].

Batch cultivation at 30 °C

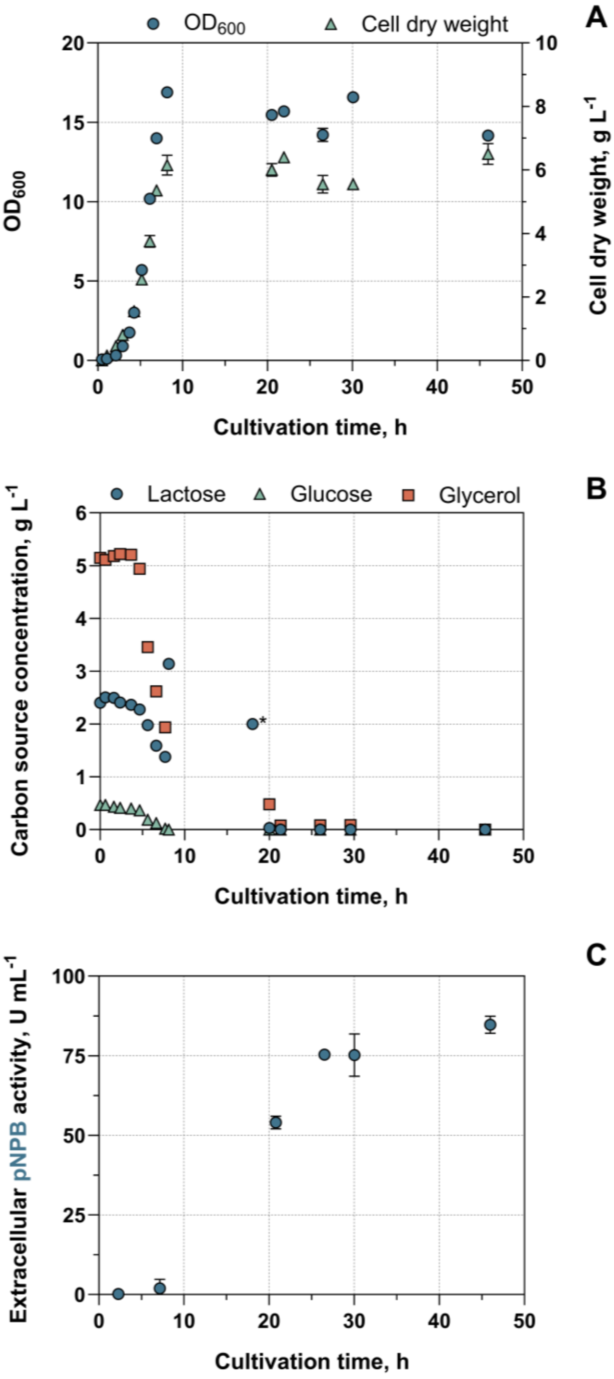

Figure S5: Batch production of ICCG$_{DAQI}$ using *E. coli* using the supplemented autoinduction media in a stirred-tank reactor at an expression temperature of 30 °C. OD_{600}, CDW (**A**) and carbon source concentrations from HPLC analysis (**B**) are shown over the cultivation time. In addition, the extracellular esterase activity using pNPB as substrate are shown (**C**). After 10 and 18 hours of growth, 2 g lactose was added. *No HPLC analysis was performed at this time, therefore the lactose concentration is presented as a theoretical value based on the addition of 2 g L^{-1}. Error bars represent the standard deviation are provided for OD_{600}, CDW and extracellular activity ($n_{technical}$ = 3), although they may be too small to be visible.

Carbon source feeding using lactose pulses

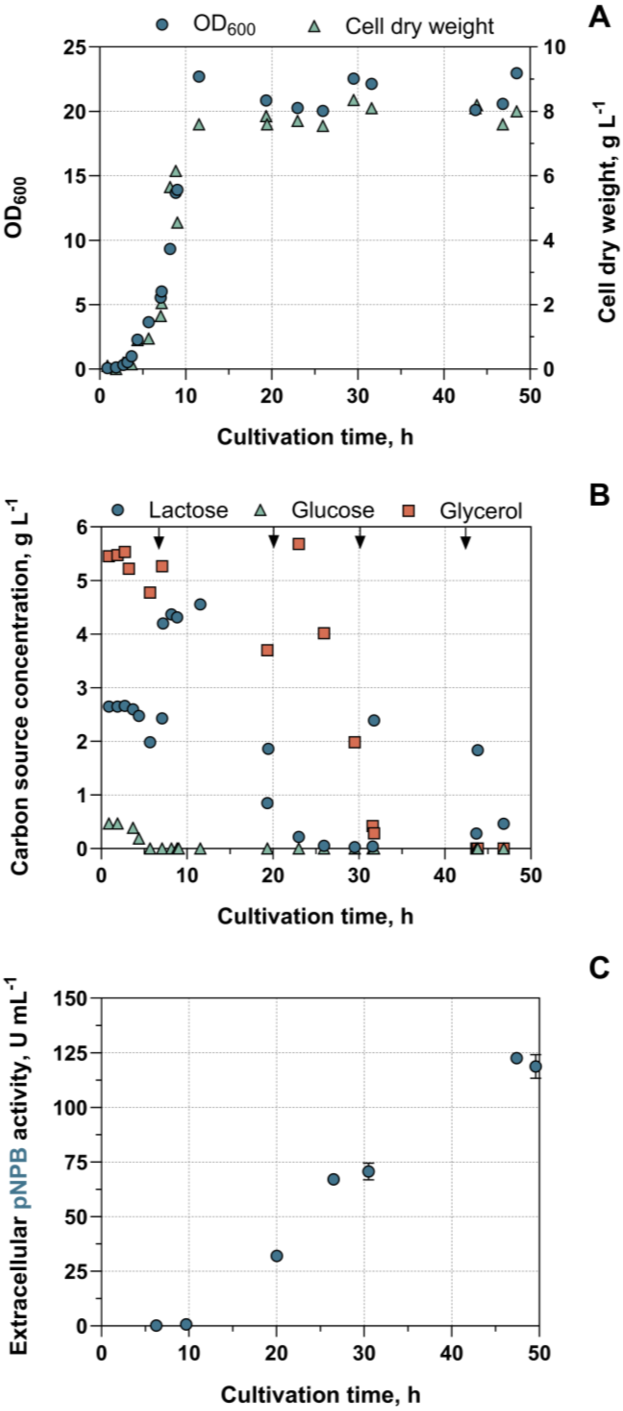

Figure S6: Batch production of $ICCG_{DAQI}$ using *E. coli* in a stirred-tank reactor at an expression temperature of 30 °C with pulsed addition of lactose. OD_{600}, CDW (**A**) and carbon source concentrations from HPLC analysis (**B**) are shown over the cultivation time. The arrows in the carbon source curves indicate a pulsed addition of lactose and glycerol (7 and 20 h) or pure lactose (30 and 44 h). In addition, the extracellular esterase activity using pNPB as substrate are shown (**C**). Error bars represent the standard deviation are provided for OD_{600}, CDW and extracellular activity ($n_{technical}$ = 3), although they may be too small to be visible.

Purification of target protein from intra- and extracellular origin

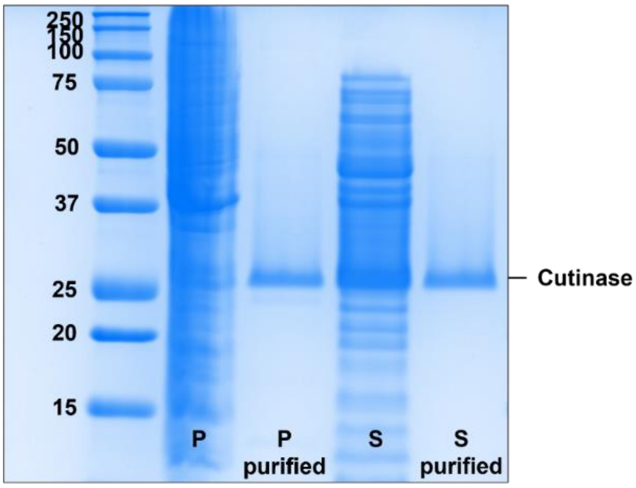

Figure S7: PAGE analysis of the cell pellet (**P**) and supernatant (**S**) as well as the corresponding eluates from IMAC purification of pellet and supernatant. After determination of the protein concentration by BCA, 2 µg protein were loaded on each lane. The bands with a calculated molecular weight of 28.4 (pellet) and 28.3 kDa (supernatant) [2] correspond to the protein of interest with a theoretical molecular weight of 28.9 kDa [3].

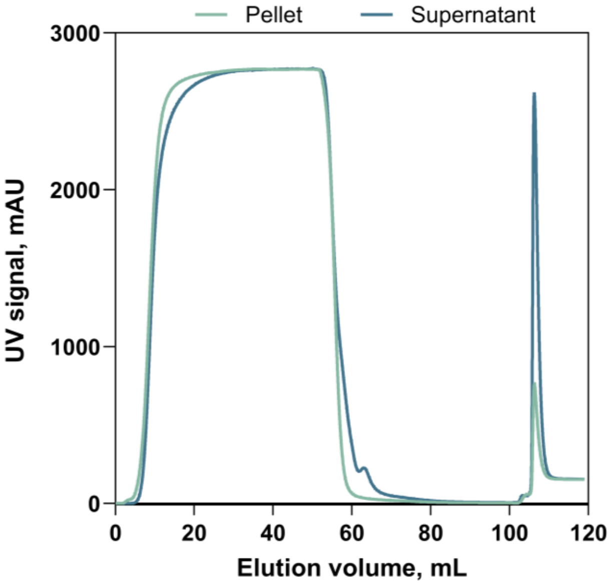

Figure S8: Chromatogram of protein purification using IMAC. After harvesting 50 mL of fed-batch *E. coli* cultivation at 30 °C, both the pellet after cell disruption (green) and the corresponding supernatant (blue) were applied to a HisTrap FF crude column. Following washing, 500 mM imidazole was used to elute the protein of interest.

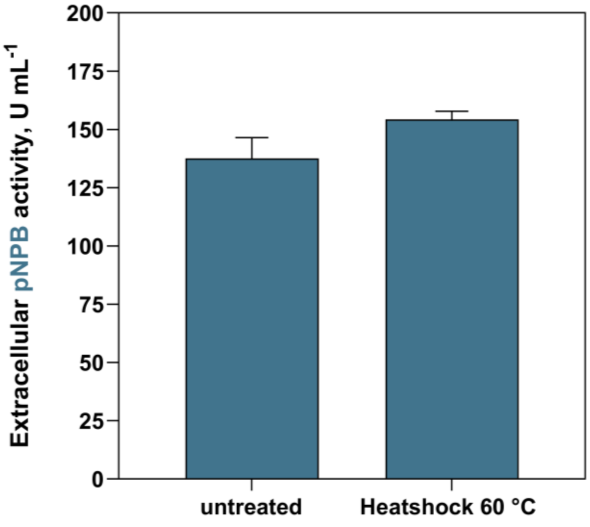

Figure S9: Treatment of the *E. coli* culture after $ICCG_{DAQI}$ at 30 °C with a heat shock at 60 °C to increase membrane permeability. The extracellular activity was determined using the pNPB assay. Each bar represents the mean ± SD of three measurements.

NMR analysis of the hydrolysis product terephthalic acid

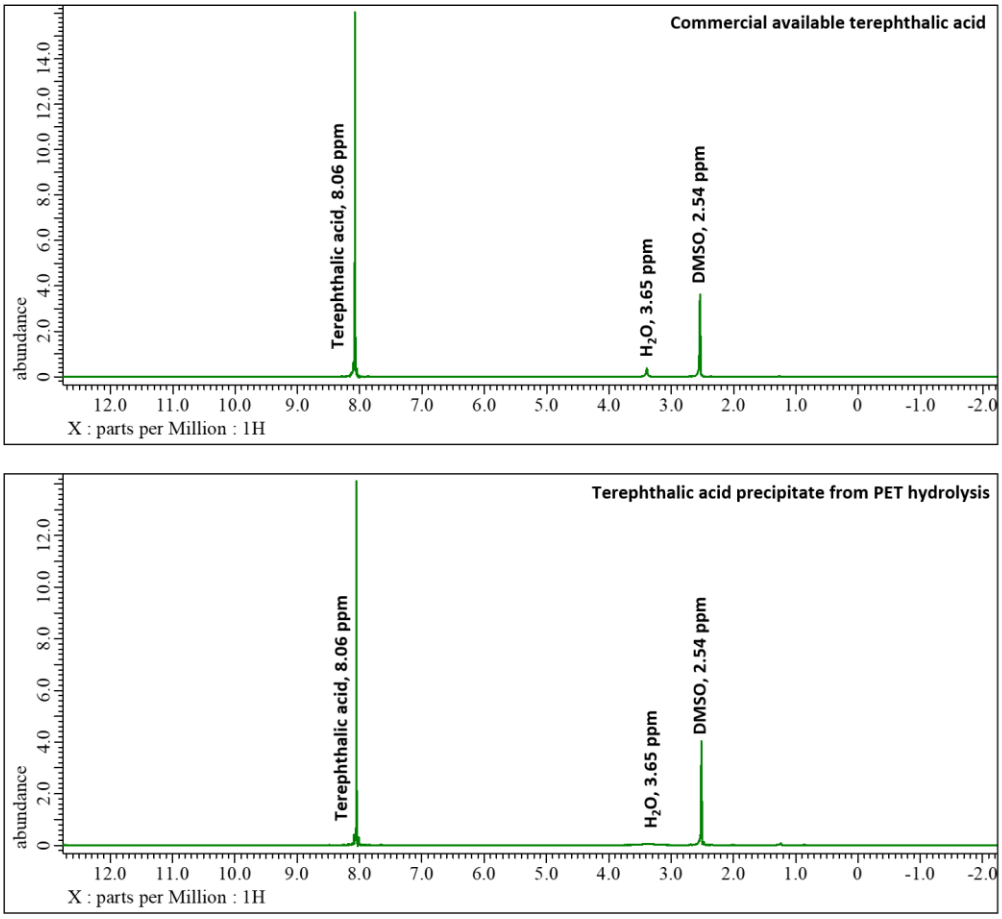

Figure S10: ^{1}H NMR spectra of commercial terephthalic acid (Thermo Fisher Scientific, Waltham, MA, >99 % purity) and the terephthalic acid precipitate (pH acidified to 2.5 with HCl) of the hydrolysis of white PET fibres using extracellular produced $ICCG_{DAQI}$. The identified compounds are labelled with the shifts given in the Spectral Database for Organic Compounds SDBS.

Protein release in the extracellular space

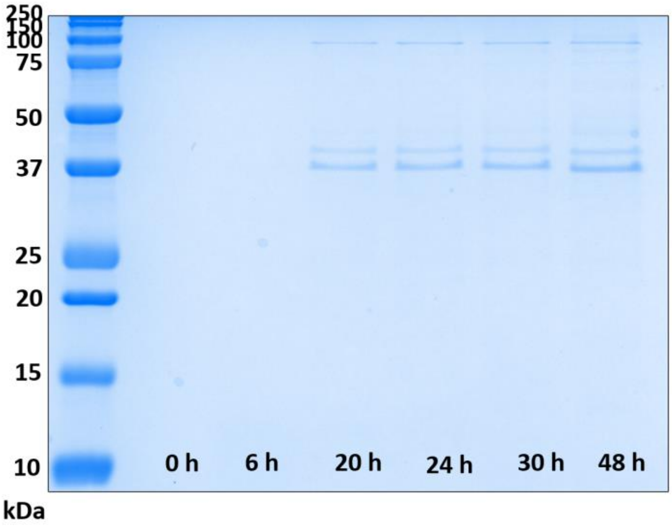

Figure S11: PAGE analysis of supernatants from an *E. coli* BL21 (DE3) culture with the empty vector (pET26b). Cultivation was performed in shake flasks with the supplemented autoinduction medium at 30 °C using the same parameters as for regular autoinduced expression.

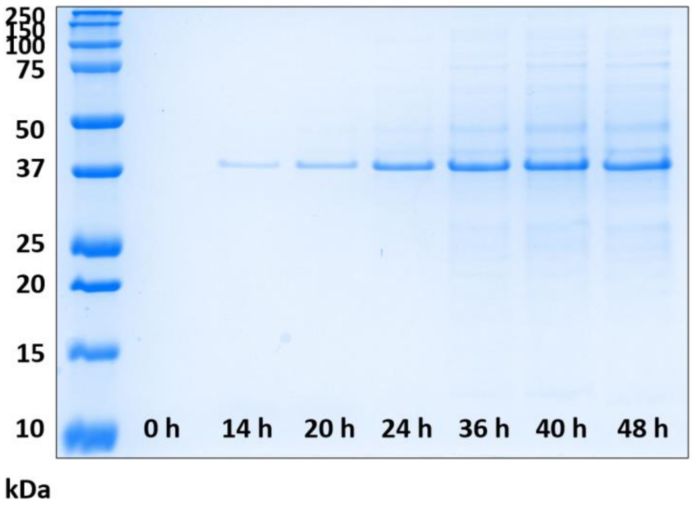

Figure S12: PAGE analysis of supernatants from an *E. coli* BL21 (DE3) culture with the empty vector (pET26b). Cultivation was performed as fed-batch in the stirred-tank reactor with supplemented autoinduction medium at 30 °C using the same parameters as for the regular fed-batch autoinduced expression.

IPTG induction

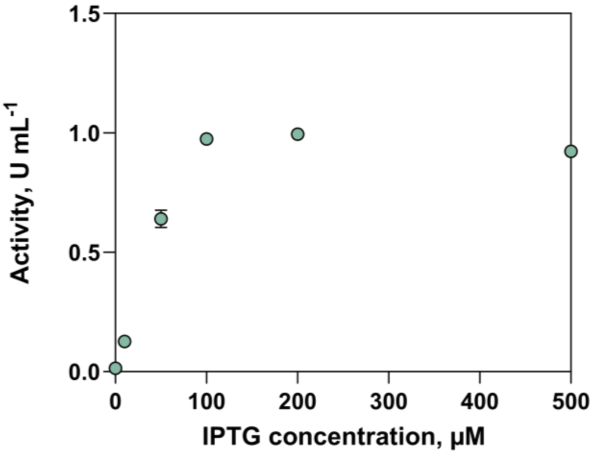

Figure S13: Cutinase activity in the supernatant over the IPTG concentration used for the induction of parallel *E. coli* cultures in shake flasks at an expression temperature of 20 °C.

References

1. Studier FW. Protein production by auto-induction in high-density shaking cultures. Protein Expr Purif. 2005;41:207–34.

2. Schneider CA, Rasband WS, Eliceiri KW. NIH Image to ImageJ: 25 years of image analysis. Nat Methods. 2012;9:671–5.

3. Gasteiger E, Hoogland C, Gattiker A, Duvaud S, Wilkins MR, Appel RD, et al. Protein Identification and Analysis Tools on the ExPASy Server. The Proteomics Protocols Handbook. Totowa, NJ: Humana Press; 2005. p. 571–607.
